# Unlocking the enigma: unraveling multiple cognitive dysfunction linked to glymphatic impairment in early Alzheimer’s disease

**DOI:** 10.3389/fnins.2023.1222857

**Published:** 2023-07-21

**Authors:** Jiayi Zhong, Xiaochen Zhang, Huanyu Xu, Xiaoran Zheng, Luyao Wang, Jiehui Jiang, Yunxia Li

**Affiliations:** ^1^School of Life Science, Shanghai University, Shanghai, China; ^2^Department of Neurology, Tongji Hospital, School of Medicine, Tongji University, Shanghai, China; ^3^School of Communication and Information Engineering, Shanghai University, Shanghai, China; ^4^Institute of Biomedical Engineering, Shanghai University, Shanghai, China

**Keywords:** Alzheimer’s disease, mild cognitive impairment, glymphatic system, diffusion tensor imaging, cognitive dysfunction, classification, medical image processing, clinical dementia rating scale

## Abstract

**Background:**

Alzheimer’s disease (AD) is one of the world’s well-known neurodegenerative diseases, which is related to the balance mechanism of production and clearance of two proteins (amyloid-β and tau) regulated by the glymphatic system. Latest studies have found that AD patients exhibit impairments to their glymphatic system. However, the alterations in the AD disease continuum, especially in the early stages, remain unclear. Moreover, the relationship between the glymphatic system and cognitive dysfunction is still worth exploring.

**Methods:**

A novel diffusion tensor image analysis method was applied to evaluate the activity of the glymphatic system by an index for diffusivity along the perivascular space (ALPS-index). Based on this method, the activity of the glymphatic system was noninvasively evaluated in 300 subjects, including 111 normal controls (NC), 120 subjects with mild cognitive impairment (MCI), and 69 subjects with AD. Partial correlation analysis was applied to explore the association between glymphatic system and cognitive impairment based on three domain-general scales and several domain-specific cognitive scales. Receiver operating characteristic curve analysis was used to evaluate the classification performance of ALPS-index along the AD continuum.

**Results:**

ALPS-index was significantly different among NC, MCI and AD groups, and ALPS-index decreased with cognitive decline. In addition, ALPS-index was significantly correlated with the scores of the clinical scales (*p*<0.05, FDR corrected), especially in left hemisphere. Furthermore, combination of ALPS and fractional anisotropy (FA) values achieved better classification results (NC vs. MCI: AUC = 0.6610, NC vs. AD: AUC = 0.8214).

**Conclusion:**

Here, we show that the glymphatic system is closely associated with multiple cognitive dysfunctions, and ALPS-index can be used as a biomarker for alterations along the AD continuum. This may provide new targets and strategies for the treatment of AD, and has the potential to assist clinical diagnosis.

## 1. Introduction

Alzheimer’s disease (AD) is a prevalent neurodegenerative disease characterized by a gradual decline in cognitive functions coupled with aberrations in mental and behavioral patterns (Livingston et al., 2020). According to the ATN framework, abnormally elevated levels of amyloid-β (Aβ) and pathological tau deposition are the main pathological features of AD (Scheltens et al., 2021; Tian et al., 2022). And these pathological features appeared in mild cognitive impairment (MCI), a pre-high-risk stage of AD (Sperling et al., 2014; Guzmán-Vélez et al., 2022). Previous studies have shown that the deposition of abnormal proteins occurs in brain regions such as neocortex and entorhinal cortex in the MCI (Duan et al., 2017; Grothe et al., 2017; Vogels et al., 2020; Berron et al., 2021). As such, investigating the mechanisms of abnormal protein production and clearance in the preclinical stages of AD could improve our understanding of the generation and development of early disease pathology and aid in prediction of cognitive decline, disease staging, and early detection (Jiang et al., 2022; Mahaman et al., 2022). To address this clearance mechanism, Iliff et al. (2012) proposed the glymphatic system, which facilitates waste removal by promoting material exchange between cerebrospinal fluid and interstitial fluid. Several animal experimental studies have shown that impaired function of the glymphatic system is associated with amyloid and tau protein deposition (Xu et al., 2015; Feng et al., 2020; Harrison et al., 2020). The mechanism of waste removal is an indispensable component in preserving a healthy brain, where the buildup of waste can cause brain damage and increase the risk of neurological diseases such as AD. It follows, therefore, that dysfunction of the glymphatic system may be a pervasive and ubiquitous phenomenon in the pre-AD stage (Nedergaard and Goldman, 2020).

The most prevalent and dependable methodology to investigate the mechanism of the glymphatic system involves the use of tracers, such as fluorescent and gadolinium-based contrast agents, in conjunction with evolving imaging techniques, including Two-Photon Imaging and Magnetic Resonance Imaging (Xie et al., 2013; Ringstad et al., 2018; Taoka et al., 2018; Taoka and Naganawa, 2020). However, the adverse effects of tracers on the human body are a problem that hinders further research (Berger et al., 2018; Edeklev et al., 2019). Recently, studies have suggested using diffusion tensor image analysis along the perivascular space (DTI-ALPS) as an alternative method. Additionally, a novel indicator, ALPS-index, has been posited to evaluate the diffusion ability of lateral white matter along the perivascular space non-invasively (Taoka et al., 2017). This method focuses on the plane of the lateral ventricles, where both the medullary veins and perivascular spaces (PVS) are oriented in a congruent spatial direction, thereby designated as the subcortical region (*X*-axis). The projection fiber, oriented perpendicular to the medullary vein (*Y*-axis) in the head-foot direction, is accompanied by the superior longitudinal fascicles (SLFs), which traverse anteriorly and posteriorly to the projection fiber (*Z*-axis) (Güngör et al., 2017). Due to the unique composition of this region, the diffusion rate of the PVS can be analyzed independently, thereby circumventing the confounding effects of diffusion along large white matter fibers. The perivascular space (PVS) represents the principal pathway of the glymphatic system. The glymphatic system functions iteratively to mitigate waste buildup by transporting cerebrospinal fluid along PVS and into the interstitial space, where metabolic waste is eliminated through material exchange (Iliff et al., 2012). Consequently, concurrent changes in the diffusion rate of the projection fiber and the associated fiber along the *X*-axis can be indicative of glymphatic system activity.

Ota et al. (2022) showed that DTI-ALPS can effectively assess the activity of the glymphatic system. In another study, it was verified that the ALPS-index is repeatable and stable across different scanning machines and imaging sequences through test–retest experiments (Taoka et al., 2022). Recently, a study reported that the glymphatic system acts as a significant mediator in AD-related cognitive dysfunction (Hsu et al., 2023). However, the change trend of the glymphatic system across the AD disease continuum, particularly in the early pathology of the disease, remains ambiguous, and further exploration is necessary to understand its relationship with cognitive decline.

In this study, we hypothesized that ALPS-index can be a potential biomarker of cognitive dysfunction along the AD continuum. The ALPS-index could be used to distinguish AD patients from normal control (NC) subjects. Studies have shown that white matter lesions are common in AD patients (Scheltens et al., 1995), which may affect the normal alterations in white matter fiber diffusivity. Based on this, before analysis, in addition to taking age, gender and education level into account, we also corrected the effect of white matter lesions. We used Analysis of Variance (ANOVA) to observe the alterations of ALPS-index along the AD continuum. Then partial correlation analysis was used to explore the relationship between ALPS-index and domain-general cognitive function scores, as well as the correlation with the four domain-specific cognitive functions (memory, execution, visual space and language). Receiver operating characteristic curve (ROC) was used to explore the potential of ALPS-index in disease diagnosis.

## 2. Materials and methods

### 2.1. Schematic of study pipeline

This study was based on a cohort from Tongji Hospital. Firstly, the images of all subjects were pre-processed using a unified process, and the diffusion parameter images were calculated. Based on the diffusion images, the ROI was located by experienced clinician, and the corresponding mask was made to extract the diffusivity in the ROI. The ALPS-index was calculated and used to assess the activity of the glymphatic system as described by Taoka et al. (2017). Subsequently, inter-group difference analysis and exploration of the glymphatic system and multiple cognitive dysfunctions were conducted by ANOVA and partial correlation analysis. Finally, ROC analysis was used to verify the clinical disease classification effect of ALPS-index.

### 2.2. Participants

This study had received approval from the Medical Research Ethics Committee at Tongji Hospital, Capital Medical University, China. In this study, we enrolled 300 subjects, including 111 NC participants, 120 MCI and 69 AD patients. All patients had signed informed consents. The concept of MCI refers to a stage between normal aging and dementia, including but not limited to subjective perception of cognitive decline and abnormal neuropsychological testing results (Petersen et al., 2001, 2009). The inclusion of AD dementia was based on clinical symptoms, with criteria derived from the Diagnostic and Statistical Manual of Mental Disorders Fifth Edition and the diagnostic guidelines for dementia due to AD established by NIA-AA workgroups (McKhann et al., 2011; Dubois et al., 2014). The detailed definitions and inclusion of AD are provided in the Supplementary material. All of the subjects were diagnosed by experienced clinicians. In this study, ALPS-index was calculated based on the white matter fibers at the lateral ventricle level of the subjects to evaluate the function of the glymphatic system. Based on this, we scored the white matter high signal of all subjects’ diffuse images by professional clinicians according to the Fazekas scale rule (Chen et al., 2022). The detailed rules of Fazekas scale are provided in the Supplementary material. Before the subsequent analysis, this scale score was regressed as a covariate to eliminate the effect of white matter lesions on the diffusion rate of normally occurring white matter fibers.

### 2.3. Cognitive function test scale

We used Minimum Mental State Examination (MMSE), Montreal Cognitive Assessment-Basic (MoCA-B) and Instrumental Activity of Daily Living (IADL) to comprehensively assess the subjects’ domain-general cognitive function. The domain-specific cognitive function in the participants is assessed using the following tools for memory, execution, visuospatial, and language function fields. The Hopkins verbal learning test-Revised (HVLT-R) is a 12-item word list and is claimed to have highly sensitive and specific for AD (Brandt, 1991; Shapiro et al., 1999). It is composed of three common categories with four words selected from each (e.g., ‘green precious stone’; ‘hotel’; ‘animal’). When the evaluator finishes reading, the subjects should recall in any order. This procedure includes immediate visual reproduction in total of three free tials and delayed recognition task (5 min and 20 min) (Shapiro and Benedict, 1999; Hogervorst et al., 2002). We used a simple executive function test: the Trail Making Test (TMT), which is widely applied in clinical practice (Toda et al., 2022). The first part, TMT-A, mainly assesses visual information processing, including searching speed and tracking (Fellows et al., 2017; Sun et al., 2020), while the second part, TMT-B, reflects working memory and cognitive flexibility (Tombaugh, 2004; Terada et al., 2013). The Rey-Osterrieth Complex Figure Test (ROCFT) was used to assess visual memory and visuoconstructional ability related to a comprehensive approach (Watanabe et al., 2005). For language measures, we used the Verbal Fluency Test (VFT) and Boston Naming Test (BNT). The former aims to evaluate a subject’s capacity to produce fluent speech and reflect other information regarding semantic memory (Olabarrieta-Landa et al., 2022). The latter consists of several drawings of common objects, and the participants are required to name them out loud for up to 20 s. This scale has been extensively used to assess confrontational naming (Ferraro et al., 1997).

### 2.4. Image acquisition and preprocessing

There were 159 subjects scanned with 3.0 T MRI scanner (SIEMENS MAGNETOM Prisma-fit). The DTI data were acquired by Gradient-echo echo-planar imaging (EPI) sequence (TR = 2,400 ms, TE = 71 ms, Slice Thickness = 2 mm, 112 × 112 matrix, 90°filp angle, voxel size = 2 × 2 × 2 mm^3^). To explore the stability of DTI-ALPS, 15 subjects underwent a secondary scan using different scanning machines and sequences. They were scanned with 3.0T MRI scanner (SIEMENS MAGNETOM Verio). The DTI data was acquired by Gradient-echo echo-planar imaging (EPI) sequence (TR = 13,700 ms, TE = 85 ms, Slice Thickness = 2 mm, 112 × 112 matrix, field of view = 224 × 224 mm^2^, 90°filp angle, voxel size = 2 × 2 × 2 mm^3^). After stability verification, 96 subjects using this scanner and sequence were also included in the study. In addition, 45 subjects were scanned with 3.0 T MRI scanner (SIEMENS MAGNETOM Verio). The DTI data was acquired by Gradient-echo echo-planar imaging (EPI) sequence (TR = 8,500 ms, TE = 98 ms, Slice Thickness = 3 mm, 74 × 74 matrix, field of view = 222 × 222 mm^2^, 90°filp angle, voxel size = 3 × 3 × 3 mm^3^).

DTI data were preprocessed using PANDA version 1.3.1.^1^ First, skull removal and cropping gap are performed to remove the unused part of the image. Next, the diffusion tensor parameters are calculated, including the diffusivity of the x, y, z axes and FA, and then the image is normalized to the Montreal Neurological Institute (MNI) template with 2 mm size. Each image is processed by applying a 6 mm Gaussian kernel smoothing and is then resampled to reduce the standardized error to improve standardization.

### 2.5. ALPS-index calculation

For subjects with three different scanning machines and parameters, 20 subjects were randomly selected from each batch. Then, the color FA map was located to the lateral ventricle plane by experienced clinicians, and ROI with a radius of 5 mm was placed in in both the projection and associated fibers. Finally, the average ROI coordinates of 20 subjects were calculated to generate the corresponding mask, to calculate the diffusion rate of all subjects in three directions (X, Y, Z). ALPS-index was calculated according to the formula proposed by Taoka et al. (2017). ROI was selected in both the left and right hemispheres of the brain and ALPS-index was calculated for each hemisphere. Based on the above, we also calculated the Bi-ALPS-index by averaging the left and right hemispheres to comprehensively evaluate the glymphatic system.


$$ALPS-index=\frac{Mean\left(D_{xxproj},D_{xxassoc}\right)}{Mean\left(D_{yyproj},D_{zzassoc}\right)}$$

### 2.6. Stability analysis of different scanning parameters

To verify whether different scanning machines and sequences in this experiment will affect the results, 15 subjects performed twice scans using different scanning machines and sequences (6 AD patients, 3 MCI patients, and 6 NC subjects). Based on this, we used the paired *t* test and intra-group correlation coefficient to verify the effects of different scanning machines and parameters.

To ensure as much as possible that ALPS-index is not affected by scanning sequences and machines, we performed batch effect correction prior to analysis, based on ComBat. ComBat is an empirical Bayes-based statistical method originally applied to genetic data and more recently widely used in neuroimaging to eliminate the effects of different scanning machines and parameters on image features (Johnson et al., 2017; Fortin et al., 2018).

### 2.7. Analysis of group difference along AD continuum

To explore changes in the ALPS-index along the AD continuum and determine if differences exist between groups, we used GraphPad Prism to conduct a one-way ANOVA. Before conducting the analysis, we adjusted for gender, age, education level, and high signal in white matter. The selection of parameters for the analysis was determined by normal distribution and homogeneity of variance tests.

Cognitive function is multidimensional, through the assessment of multiple functional areas. In view of this, in order to explore whether the activity of the glymphatic system can characterize the cognitive function of AD patients as comprehensively as possible. We first analyzed the correlation between ALPS-index and three clinical domain-general cognitive scales (MoCA-B, MMSE, IADL). On this basis, we further explored the correlation with domain-specific cognitive functions. Ten cognitive scales were used to evaluate the four cognitive domain functions of memory, execution, visual space and language.

### 2.8. Disease classification

We use the support vector machine (SVM) model to test whether the ALPS-index has the ability to distinguish AD from other groups. The ALPS-index of the left, right and whole brain was used as the feature input, and the traditional DTI parameter FA was input for comparison. In the process of model construction, the ratio of training set to test set was 4:1, and 5-fold cross-validation was used to avoid model over-fitting. The stability of the model is maintained by averaging 100 cycles of training. The ROC curve was drawn based on the results on the test set. We conducted classification experiments on NC group and MCI group, MCI group and AD group, NC group and AD group, respectively.

### 2.9. Statistical analysis

Demographic information was analyzed using GraphPad Prism version 9.0.0 for Windows (GraphPad Software, San Diego, California, United States)^2^, and One-way ANOVA was conducted. Partial correlation analysis was performed using SPSS version 26.0 (IBM Corp, Armonk, NY, United States). MATLAB was utilized in constructing and validating the classification model. A significance level of *p* < 0.05 was chosen. The multiple comparison test utilized the Benjamini-Hochberg False Discovery Rate (BH-FDR) test.

## 3. Results

### 3.1. Demographic data

Table 1 shows the demographic data of 300 patients. There was no significant difference in gender among the three groups (*p* = 0.713). The average age of the three groups was statistically significant (*p* < 0.0001), among which the average age of AD patients was the highest (74 years old). The table also indicates significant differences in education level among the three groups (*p* < 0.0001), with NCs having the highest mean education level. We found that there was a significant difference in white matter high signal between the three groups (*p* = 0.0001), and the mean score showed an upward trend. Subsequent studies eliminated its effects by considering it as a covariate. Moreover, significant differences were observed in all scale scores among the three groups (*p* < 0.0001), indicating that the study groups were distinct clinically.

**Table 1:** Participants and clinical characteristics.

	NC	MCI	AD	*P*
Gender	54:57	52:68	31:38	0.713
Age	68.6 ± 7.5	72.0 ± 7.1	74.0 ± 8.5	<0.0001
Edu	12.2 ± 3.6	10.0 ± 4.4	9.4 ± 5.2	<0.0001
WMH	2.0 ± 1.5	2.5 ± 1.5	3.1 ± 1.6	0.0001
MoCA-B	23.8 ± 3.7	16.9 ± 3.8	7.9 ± 3.6	<0.0001
MMSE	27.0 ± 2.2	24.2 ± 2.9	14.9 ± 5.8	<0.0001
IADL	15.2 ± 2.6	16.7 ± 2.9	24.1 ± 6.4	<0.0001
HVLT1	20.5 ± 4.5	15.3 ± 4.2	7.4 ± 4.6	<0.0001
HVLT2	7.2 ± 2.3	3.0 ± 2.9	0.2 ± 1.0	<0.0001
HVLT3	7.3 ± 2.2	2.8 ± 2.8	0.3 ± 0.9	<0.0001
WMS	9.5 ± 2.4	6.2 ± 2.3	3.0 ± 2.3	<0.0001
TMT-A	54.7 ± 16.7	76.6 ± 28.0	120.0 ± 48.8	<0.0001
TMT-B	139.6 ± 44.4	180.1 ± 51.5	211.7 ± 50.3	<0.0001
ROCFT1	25.2 ± 13.3	23.9 ± 12.6	15.1 ± 12.9	<0.0001
ROCFT2	13.4 ± 8.7	6.7 ± 6.8	0.9 ± 2.8	<0.0001
VFT	14.7 ± 3.5	11.6 ± 3.2	6.3 ± 3.3	<0.0001
BNT	23.9 ± 3.4	19.9 ± 4.2	14.2 ± 5.7	<0.0001

Gender is presented as Male: Female, use chi-square test. Age, Edu, WMH and all cognitive scale scores are presented as mean ± standard deviation, use Analysis of Variance. HVIT1 was immediate visual reproduction test, HVIT2 and HVIT3 were delayed visual tests (5 min and 20 min), respectively. ROCFT1 represents copying figure test, ROCFT2 represents memory figure test.

### 3.2. The influence of scanning machine and sequence

**Figure 1:**
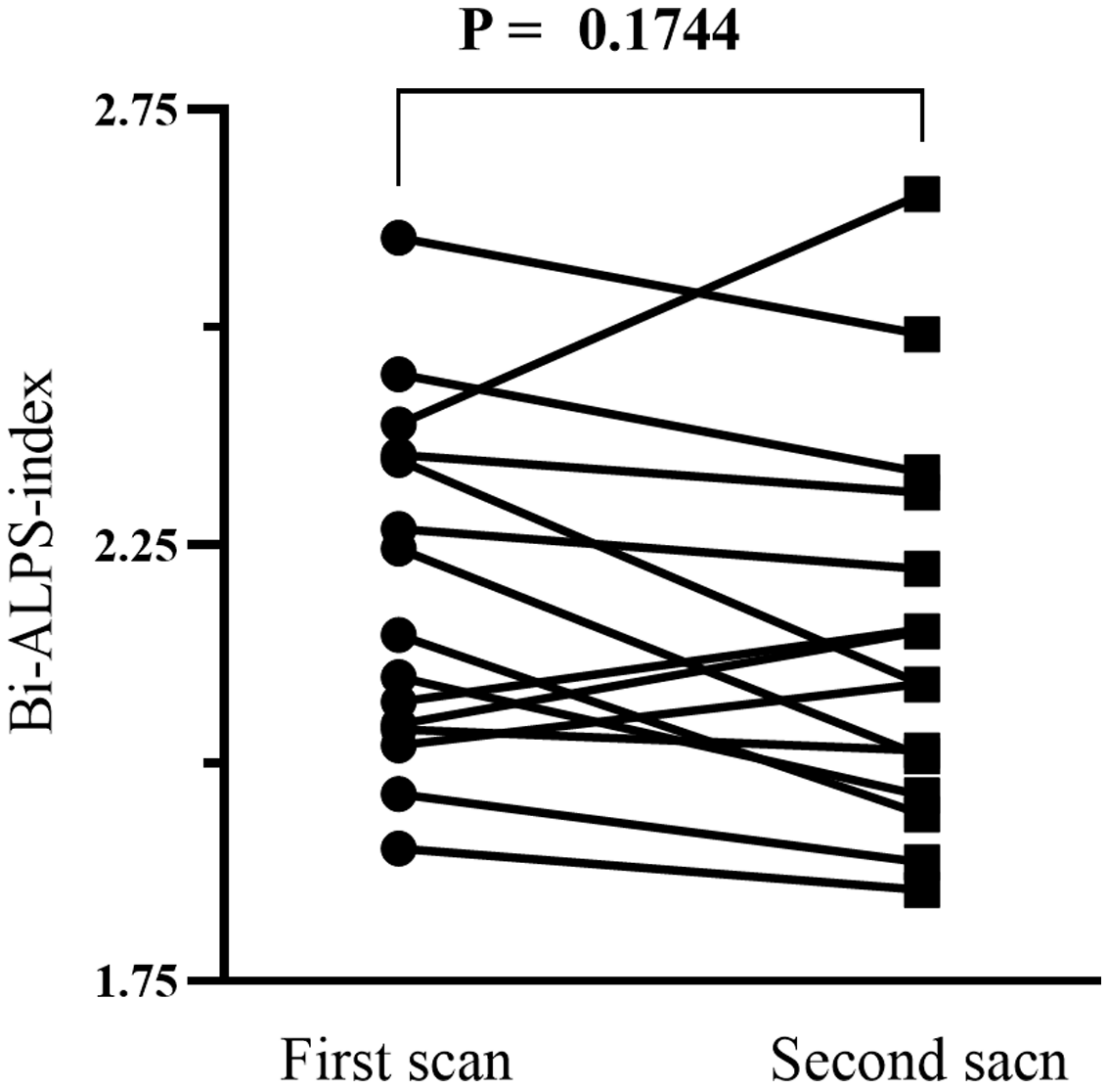
Paired *t* test results of different scanning machines and sequences.

In the stability experiment, the ALPS-index calculated by two scans showed high stability. Figure 1 shows the results of paired *t* test. There was no significant difference in Bi-ALPS-index between the two groups (*p* = 0.1744). Then through the analysis of intra-group correlation coefficient we found high similarity between two scans (ICC = 0.7723).

### 3.3. Alterations of ALPS-index along the AD continuum

**Figure 2:**
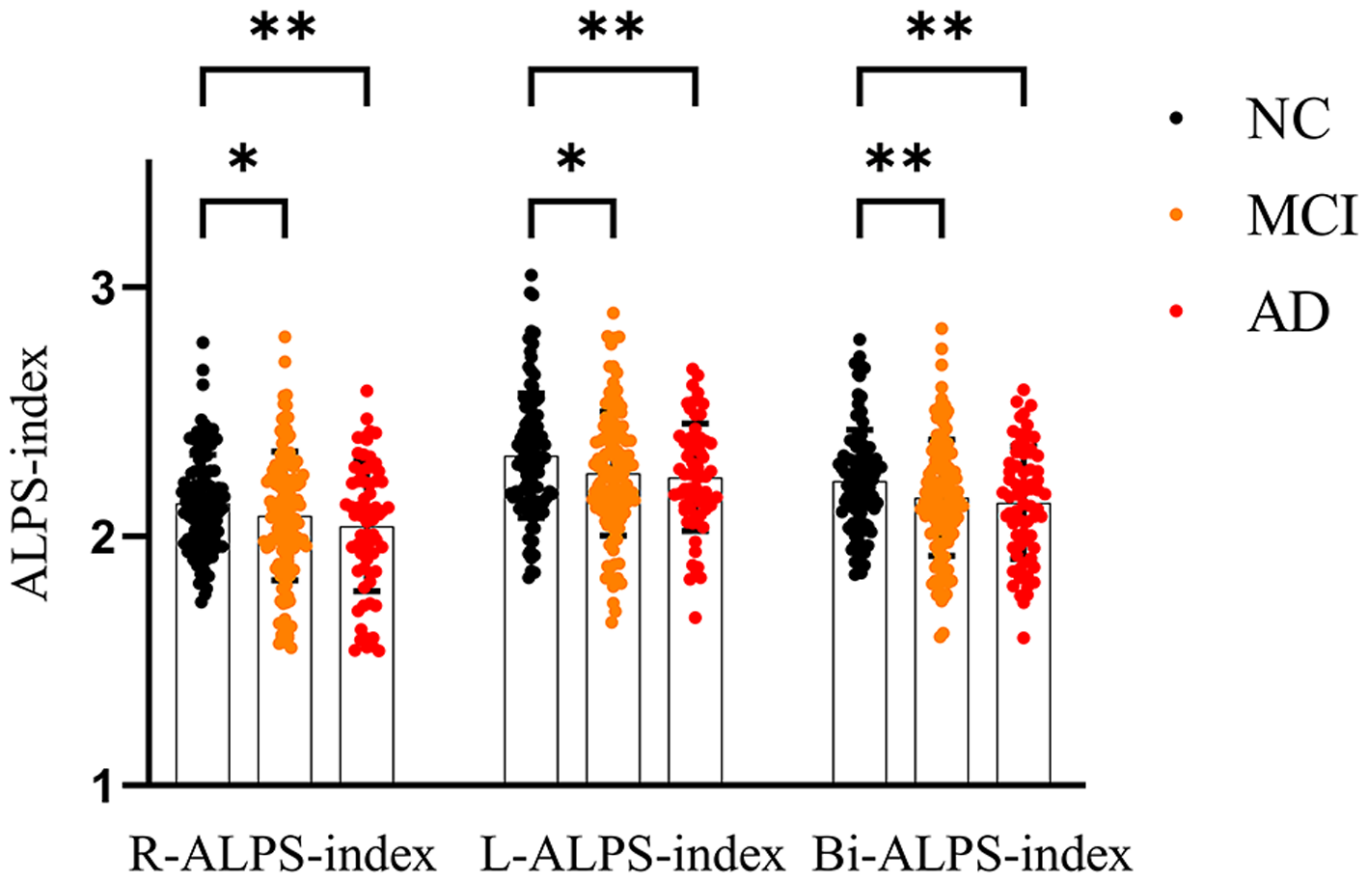
One-way ANOVA results among NC, MCI and AD groups. Bi, L and R represent the mean values of right brain, left brain and left and right brain, respectively. *p < 0.05, **p < 0.01.

Along the AD continuum, ALPS-index was on the decline and was the lowest in the AD group (Figure 2). After adjustment for covariates, there were significant differences between NC and AD groups in all three diffusion indexes (R-ALPS-index, L-ALPS-index and Bi-ALPS-index). Furthermore, there were significant differences between NC and MCI in all three diffusion indexes, with the strongest significance in Bi-ALPS-index (*p* < 0.01). But no significant differences were observed between MCI and AD groups.

### 3.4. Correlation between ALPS-index and clinical scales

**Figure 3:**
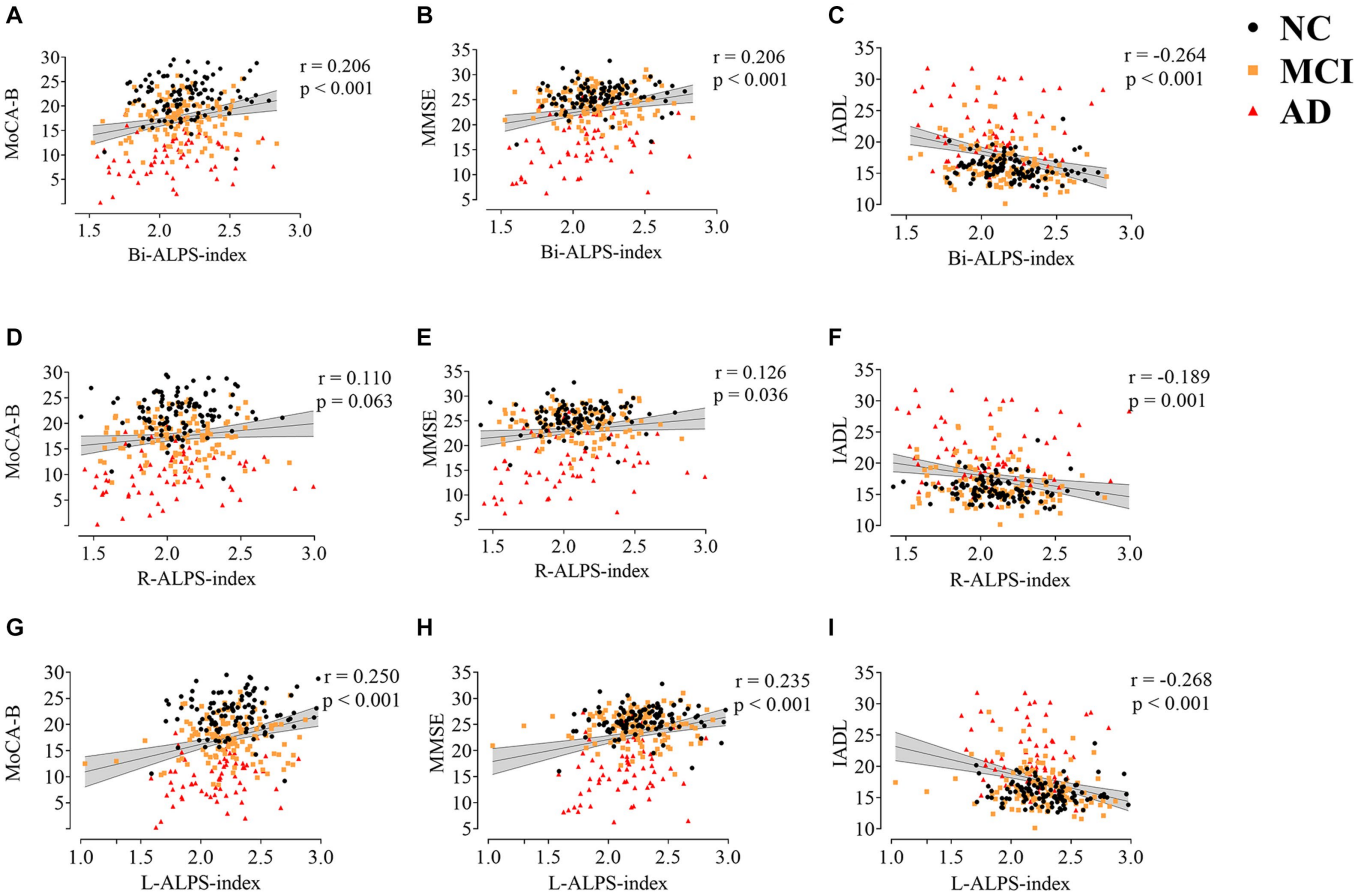
The correlation between ALPS-index (Bi,R,L) and MoCA-B **(A,D,G)**, MMSE **(B,E,H)**, and IADL **(C,F,I)**. MoCA-B, Montreal Cognitive Assessment-Basic; MMSE, Minimum Mental State Examination; IADL, Instrumental Activity of Daily Living.

In order to explore the relationship between the glymphatic system and cognitive dysfunction, we first analyzed the correlation between ALPS-index and domain-general cognitive scale. Figure 3 shows the results of the correlation analysis between ALPS Indexes and MoCA-B, MMSE, and IADL after correction for age, WMH, and education. There is a significant correlation between L-ALPS-index and Bi-ALPS-index and the three scales. There was a significant positive correlation with MoCA-B (*r* = 0.250, *p* < 0.001; *r* = 0.206, *p* < 0.001) and MMSE (*r* = 0.235, *p* < 0.001; *r* = 0.206, *p* < 0.001), and a significant negative correlation with IADL (*r* = −0.268, *p* < 0.001; *r* = −0.264, *p* < 0.001). The R-ALPS-index was found to be significantly negatively correlated with IADL (*r* = −0.189, *p* = 0.001), and a significant positive correlation with MMSE (*r* = 0.126, *p* = 0.036), but was not found to be significantly negatively correlated with MoCA-B (*r* = 0.110, *p* = 0.063).

**Figure 4:**
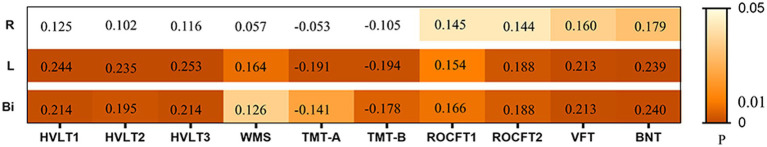
Heat map and correlation between ALPS-index and domain -specific cognitive function test.

For domain-special cognitive function, Figure 4 shows the correlation between ALPS-index and 10 cognitive tests after adjusting for gender, age, education level, and white matter high signal. L-ALPS-index and Bi-ALPS-index were significantly correlated with the 10 domain-specific cognitive scales (HVLT1, HVLT2, HVLT2, WMS, ROCFT1, ROCFT2, VFT and BNT), positive correlations, and significant negative correlations with TMT-A and TMT-B (see Figure 4). However, significant correlations were only found with ROCFT, ROCFT2, VFT and BNT in R-ALPS-index.

### 3.5. Classification experiments

**Figure 5:**
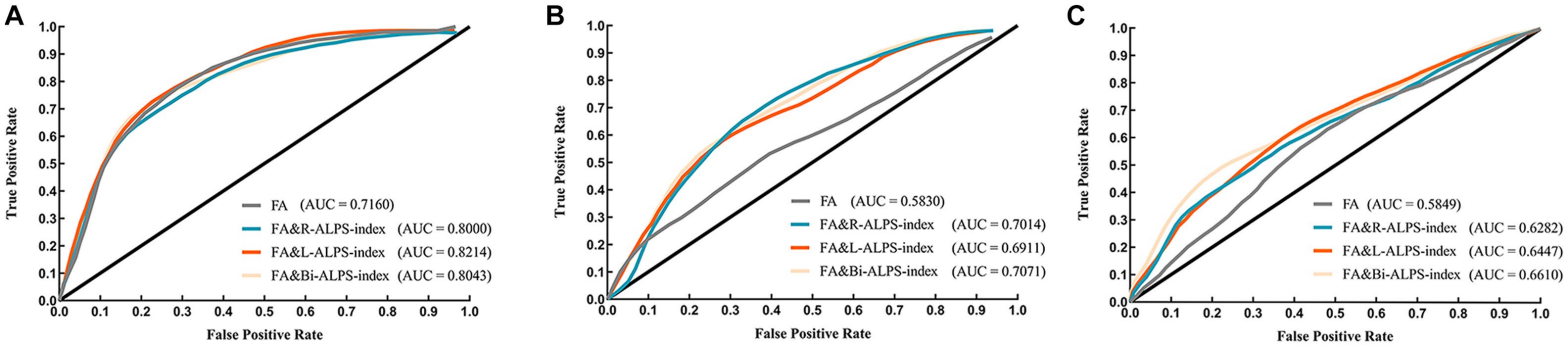
The ROC curves of classifier in different groups. **(A)** NC and AD groups. **(B)** MCI and AD groups. **(C)** NC and MCI groups.

We evaluated the classification performance of ALPS-index in the AD disease continuum. Firstly, we used FA to classify NC, MCI and AD. In the classification experiments, the AUC values of FA classification were 0.7160, 0.5562, and 0.5849, respectively. When we use ALPS-index and FA for joint classification, the classification effect has been significantly improved (Figure 5). In the classification of NC and AD groups (Figure 5A), combined with FA and ALPS-index (R, L, Bi), the AUC values (95% CI) reached 0.8000 (0.7936 to 0.8063), 0.8214 (0.8184 to 0.8300), and 0.8043 (0.8013 to 0.8144). When classifying MCI and AD groups (Figure 5B), the AUC value reached to 0.7014 (0.6824 to 0.7169), 0.6911 (0.6897 to 0.7045), 0.7071 (0.6718 to 0.7161) by ALPS-index (R, L, Bi) combined classification. When distinguishing MCI and NC groups (Figure 5C), the results show that ALPS-index also improves the classification effect, and the highest value of AUC was achieved to 0.6610 (0.6523 to 0.6717).

## 4. Discussion

We explored the alterations of the glymphatic system along the AD continuum through ANOVA and partial correlation. ALPS-index was used to evaluate the activity of the glymphatic system. We found that along the AD continuum, ALPS-index showed a downward trend, which was significantly correlated with domain-general cognitive function scores. Furthermore, we found that ALPS-index was significantly correlated with several domain-specific cognitive tests. In the classification experiment, FA combined with ALPS-index achieved better classification results for NCs and ADs. The present findings suggest that impairment of the glymphatic system is associated with multiple cognitive impairments. This suggests that the glymphatic system plays a role in the detection of cognitive decline as well as the early diagnosis of AD.

In our study, we first verified the reproducibility of ALPS-index, that is, ALPS-index obtained when subjects had different scanners and sequences had a high ICC value (0.7723). DTI-ALPS is a method proposed by Taoka et al. (2017) to evaluate the activity of the glymphatic system. The reproducibility of ALPS-index was similarly validated in their studies (Taoka et al., 2022). They first found a decrease in ALPS-index in patients with AD (Taoka et al., 2017), similar to our results. Furthermore, we further explored the trend of ALPS-index along the AD disease continuum, and the results showed that the downward trend started from the MCI stage. A recent study (Steward et al., 2021) measured DTI-ALPS along the perivascular space in 10 normal controls, 10 people with mild cognitive impairment and 16 people with AD. After controlling for age and gender effects, they found there was a significant difference in R-ALPS-index between NC group and AD group, but not in the left hemisphere. Compare that to our study, we also found that R-ALPS-index was significantly different between AD and NC groups. In contrast, we also found the same results in the left hemisphere. As mentioned by Steward et al., this may be due to white matter lesions were not considered in their study, which may affect the normal diffusivity changes of the fibers. We also did an analysis that did not eliminate white matter lesions, and found no significant difference between the three groups in the R-ALPS index (see Supplementary Figure S1), but the downward trend still exists in the AD disease continuum. In addition, the correlation and significance between ALPS index and various cognitive scales also decreased (see Supplementary Figures S2, S3). Therefore, white matter lesions have a potential effect on the ALPS-index. Moreover, in our study, we found a significant difference in ALPS-index between MCI and NC groups, indicating that the glymphatic system is impaired in MCI stage. These results suggest that ALPS-index may serve as a tool for early diagnosis. Although the same results were not found between MCI and AD groups, it can also be seen that there is a downward trend between MCI and AD groups.

Some previous DTI-ALPS studies have shown that ALPS-index was significantly positively correlated with MMSE scores, and it has been found that the ALPS-index of AD patients was reduced, indicating that the activity of the glymphatic system was damaged (Taoka et al., 2017; Ma et al., 2021; Steward et al., 2021). This is consistent with our findings. In addition, we further found that ALPS-index was positively correlated with MoCA-B score and negatively correlated with IADL score. MoCA-B is more sensitive than MMSE in screening early AD (Ciesielska et al., 2016). This further increases the potential of ALPS-index as a marker for early AD screening.

To further explore the relationship between glymphatic system activity and cognitive impairment, we explored the correlation between ALPS-index and 10 domain-specific cognitive scales. Even after correcting the effects of multiple covariates including white matter hyperintensity, we found that ALPS-index was significantly correlated with HVLT, VFT, ROCFT, TMA and BNT. This suggests that the glymphatic system is involved in these cognitive impairments: memory, executive function, visuospatial, and language. Recent studies on the association of the glymphatic system with abnormal protein deposition based on PET images support our results. Ota et al. (2022) found that ALPS-index was significantly correlated with deposition of abnormal protein (Aβ and tau) in bilateral temporal lobe cortex, left and right parietal lobe cortex and posterior cingulate gyrus. Another study considered the effect of AD-related gray matter ratio, and still found ALPS-index was a significant mediator of the relationship between abnormal protein deposition and cognitive impairment in multiple brain regions (Hsu et al., 2023). The above findings indicate that the glymphatic system is active in these brain regions, which are responsible for cognitive functions such as memory, execution, visuospatial and language. This could provide insight into the relationship between brain waste removal and cognitive decline, as well as provide a new direction for researchers studying cognitively related brain structures and regions. There are few studies focused on the effect of ALPS-index disease classification. We construct SVM classification model and use ROC curve to evaluate the classification effect of ALPS-index. The results of ROC show that ALPS-index and FA can achieve better classification results. When distinguishing AD and NC groups, the highest AUC was 0.82147. However, when distinguishing MCI from AD and MCI from NC, the AUC value is low, only around 0.6–0.7.

However, it is worth noting that there are still some limitations in the current research. First of all, though we control the influence of multiple variables including white matter lesions, but there may still be some influential factors for classes the glymphatic system activity. Secondly, the role and mechanism of the activity of the glymphatic system in the relationship between Aβ and tau deposition and cognitive function need to be further explored. Finally, a larger sample size and multi-center data are expected to verify these results in the future.

## 5. Conclusion

Based on these experimental results, we suggest that ALPS-index can be a marker of cognitive dysfunction and with significant decline in the MCI stage, indicating that it may be a marker for early diagnosis. In partial correlation analysis, our results also indicate that the glymphatic system is closely associated with multiple cognitive scales. Our results suggest that the glymphatic system may be a new target for cognitive decline, early detection, and treatment of AD.

## Data availability statement

The original contributions presented in the study are included in the article/Supplementary material, further inquiries can be directed to the corresponding authors.

## Ethics statement

The studies involving human participants were reviewed and approved by the Medical Research Ethics Committee at Tongji Hospital, Capital Medical University, China. The patients/participants provided their written informed consent to participate in this study.

## Author contributions

JZ and XZhang: investigation, methodology, specific experiment, and wrote the original draft. HX and XZheng: data curation, formal analysis, and methodology. LW, YL, and JJ: conceptualization, guide, and provide unique insights in experiments and revised draft. All authors contributed to the article and approved the submitted version.

## Funding

This work was supported by the Shanghai Science and Technology Development Foundation (Sailing Program) (22YF1413900), the National Natural Science Foundation of China (Grant 82020108013, 81871438, 820017773, and 62206165), and the Research project of Shanghai Health Commission (2020YJZX0111).

## Conflict of interest

The authors declare that the research was conducted in the absence of any commercial or financial relationships that could be construed as a potential conflict of interest.

## Publisher’s note

All claims expressed in this article are solely those of the authors and do not necessarily represent those of their affiliated organizations, or those of the publisher, the editors and the reviewers. Any product that may be evaluated in this article, or claim that may be made by its manufacturer, is not guaranteed or endorsed by the publisher.

## Footnotes

^1^https://www.nitrc.org/projects/panda/

^2^www.graphpad.com
