# Supplementary material for: Unlocking the enigma: unraveling multiple cognitive dysfunction linked to glymphatic impairment in early Alzheimer’s disease
Source: Front Neurosci. 2023 Jul 21;17:1222857. doi: 10.3389/fnins.2023.1222857 (PMC10400773; doi:10.3389/fnins.2023.1222857)
Supplement: Supplementary file 1 [file Data_Sheet_1.docx]

Supplementary Material

**Unlocking the Enigma: Unravelling Multiple Cognitive Dysfunction Linked to Glymphatic Impairment in Early Alzheimer's Disease**

**Jiayi Zhong ^1†^, Xiaochen Zhang^3†^,** **Huanyu Xu^2^, Xiaoran Zheng^3^, Luyao Wang^1^*, Jiehui Jiang^4^*, Yunxia Li^3^***

*** Correspondence:**

Luyao Wang: wangly1018@shu.edu.cn

Yunxia Li*

liyunxiadoctor@163.com

Jiehui jiang*

jiangjiehui@shu.edu.cn

# Detailed definitions of AD

Firstly, all the patients should meet criteria for dementia described in the following, interfere with the ability to function at work or at usual activities and represent a decline from previous levels of functioning and performing, moreover, cognitive impairment is detected and diagnosed through a combination of history-taking from the patient and a knowledgeable informant and an objective cognitive assessment, either a “bedside” mental status examination or neuropsychological testing. Neuropsychological testing should be performed when the routine history and bedside mental status examination cannot provide a confident diagnosis.

Secondly, the probable AD dementia has the following characteristics: (1) Symptoms have a gradual onset over months to years, not sudden over hours or days; (2) Clear-cut history of worsening of cognition by report or observation; (3) The initial and most prominent cognitive deficits are evident in one of the following categories: ①Amnestic presentation, the most common syndromic presentation of AD dementia, should include impairment in learning and recall of recently learned information. There should also be evidence of cognitive dysfunction in at least one other cognitive domain. ②Nonamnestic presentations: language presentation, the most prominent deficits are in word-finding, but deficits in other cognitive domains should be present; visuospatial presentation, the most prominent deficits are in spatial cognition, including object agnosia, impaired face recognition, simultanagnosia, and alexia; executive dysfunction, the most prominent deficits are impaired reasoning, judgment, and problem solving. A diagnosis of possible AD dementia should be made in either of the circumstances mentioned in the following paragraphs.

Thirdly, a diagnosis of possible AD dementia should be made in either of the circumstances mentioned in the following: (1) Atypical course. The atypical course meets the core clinical criteria in terms of the nature of the cognitive deficits for AD dementia, but either has a sudden onset of cognitive impairment or demonstrates insufficient historical detail or objective cognitive documentation of progressive decline; (2) Etiologically mixed presentation. The etiologically mixed presentation meets all core clinical criteria for AD dementia but has evidence of (a) concomitant cerebrovascular disease, defined by a history of stroke temporally related to the onset or worsening of cognitive impairment; or the presence of multiple or extensive infarcts or severe white matter hyperintensity burden; or (b) features of Dementia with Lewy bodies other than the dementia itself; or (c) evidence for another neurological disease or a non-neurological medical comorbidity or medication use that could have a substantial effect on cognition.

# Fazekas scale for white matter lesions

White matter lesions are graded using the rules of the Fazekes scale, which is done by an experienced physician. Here are the scoring rules:

Periventricular hyperintensity (PVH) was graded as 0=absence, 1= “caps” or pencil-thin lining, 2= smooth “halo”, 3=irregular PVH extending into the deep white matter.

Separate deep white matter hyperintense signals (DWMH) were rated as 0=absence, 1=punctate foci, 2=beginning confluence of foci, 3=large confluent areas.

Then, the PVH and DWMH scores were added together as the white matter high signal of the subjects.

# Supplementary Figures and Tables

## Supplementary Figures 1

**
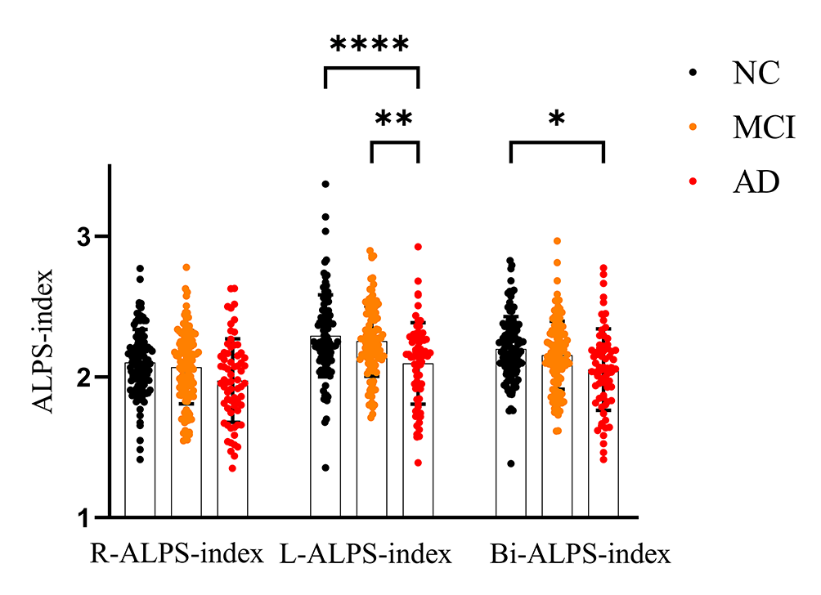
**

**Supplementary Figure 1.** ANOVA results that did not eliminate the effects of white matter lesions. Bi, L and R represent the mean values of right brain, left brain and left and right brain respectively. * p < 0.05, * * p < 0.01.

## Supplementary Figures 2

#
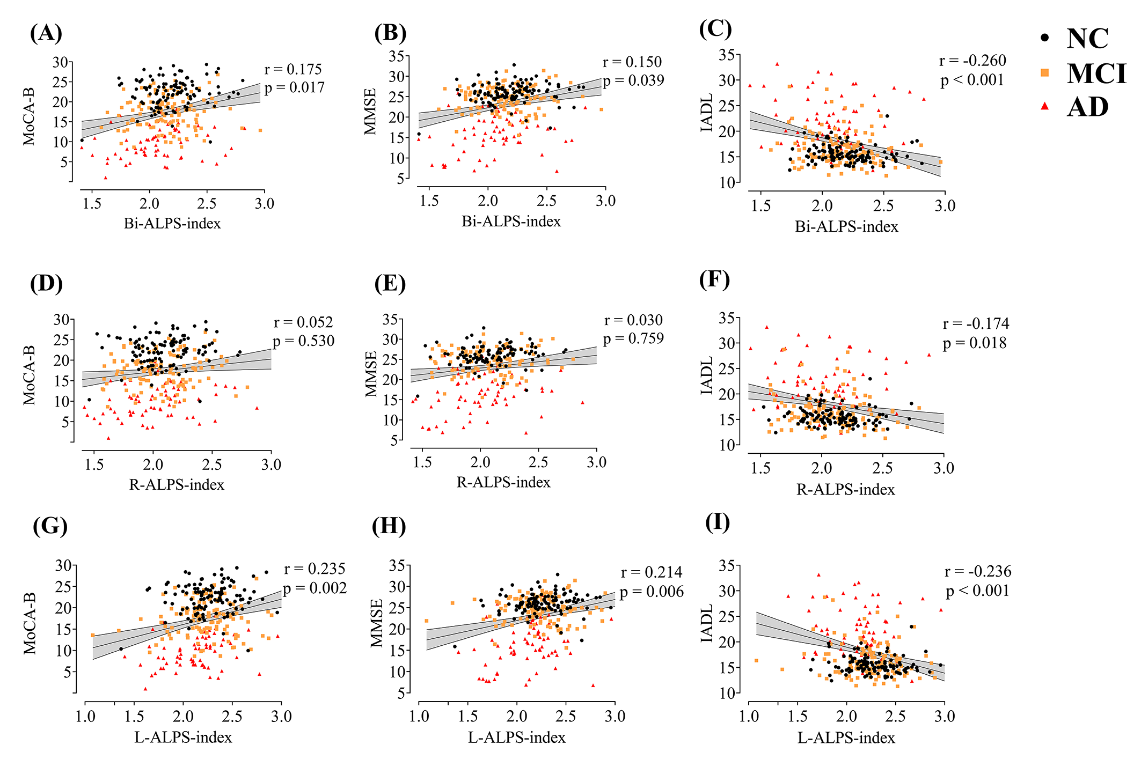


**Supplementary Figure 2.** This is the result of not doing anything to eliminate the effects of white matter disease. The correlation between Bi-ALPS-index and (A) MoCA-B, (B) MMSE and (C) IADL. MoCA-B: Montreal Cognitive Assessment-Basic, MMSE: Minimum Mental State Examination, IADL: Instrumental Activity of Daily Living. 95 % confidence interval.

## Supplementary Figures 3

#
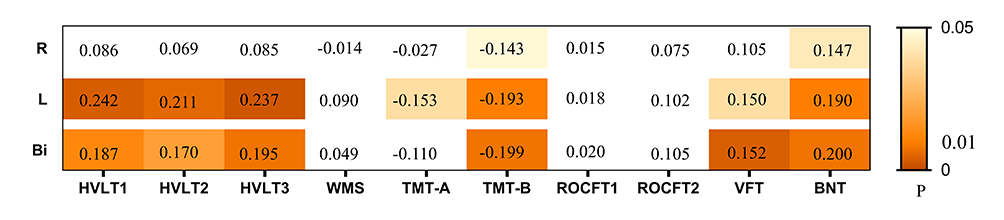


**Supplementary Figure 3.** This is the result of not doing anything to eliminate the effects of white matter disease. Heat map and correlation between ALPS-index and domain -specific cognitive function test.
